# Automated Analysis of Two-Dimensional Positions and Body Lengths of Earthworms (*Oligochaeta*); MimizuTrack

**DOI:** 10.1371/journal.pone.0097986

**Published:** 2014-06-02

**Authors:** Naomi Kodama, Toshifumi Kimura, Seiichiro Yonemura, Satoshi Kaneda, Mizue Ohashi, Hidetoshi Ikeno

**Affiliations:** 1 National Institute for Agro-Environmental Sciences (NIAES), 3-1-3 Kannondai, Tsukuba, Ibaraki, Japan; 2 School of Human Science and Environment, University of Hyogo, 1-1-12 Shinzaike-honcho, Himeji, Hyogo, Japan; University of Western Australia, Australia

## Abstract

Earthworms are important soil macrofauna inhabiting almost all ecosystems. Their biomass is large and their burrowing and ingestion of soils alters soil physicochemical properties. Because of their large biomass, earthworms are regarded as an indicator of “soil heath”. However, primarily because the difficulties in quantifying their behavior, the extent of their impact on soil material flow dynamics and soil health is poorly understood. Image data, with the aid of image processing tools, are a powerful tool in quantifying the movements of objects. Image data sets are often very large and time-consuming to analyze, especially when continuously recorded and manually processed. We aimed to develop a system to quantify earthworm movement from video recordings. Our newly developed program successfully tracked the two-dimensional positions of three separate parts of the earthworm and simultaneously output the change in its body length. From the output data, we calculated the velocity of the earthworm's movement. Our program processed the image data three times faster than the manual tracking system. To date, there are no existing systems to quantify earthworm activity from continuously recorded image data. The system developed in this study will reduce input time by a factor of three compared with manual data entry and will reduce errors involved in quantifying large data sets. Furthermore, it will provide more reliable measured values, although the program is still a prototype that needs further testing and improvement. Combined with other techniques, such as measuring metabolic gas emissions from earthworm bodies, this program could provide continuous observations of earthworm behavior in response to environmental variables under laboratory conditions. In the future, this standardized method will be applied to other animals, and the quantified earthworm movement will be incorporated into models of soil material flow dynamics or behavior in response to chemical substances present in the soil.

## Introduction

Earthworms, the so-called ecosystem engineers, are important soil macrofauna belonging to the subclass *Oligochaeta*. They inhabit various types of soil ranging from 10 to 2000 individuals per meter squared, resulting in a large biomass of between 0.5 to 305 g dry mass m^−2^ [1]. They significantly change soil properties and fertility in the vicinity of the large burrowing areas [2]. Their important role in soil formation has been already emphasized by Darwin [3]. Earthworms are one of the main components of the drilosphere, one of the biological systems regulating mineralization of soil organic matter, where they activate microbes by mixing the litter and soil, breaking down the physical protection of soil organic matter, and developing soil aggregates during gut transit [2]. The changes in soil pore structures and the extent of aggregations also alter soil gas diffusion coefficients physically, which could increase emissions of global warming gases, such as CO_2_, CH_4_ and N_2_O [4]. Because of their behaviors and features, earthworms are regarded as important biological indicators of chemical toxicity in soil ecosystems and many studies have examined the effects on population dynamics of earthworms such as fecundity, reproductive activity, and mortality [5]. Their behaviors affect not only below-ground material flow dynamics, but also indicate “soil health” conditions.

Evaluation of the functional role of earthworms on soil ecosystems has been, however, insufficient because of methodological difficulties [6]. Traditional research methods including descriptive observations of their behavior, gut content analysis, choice tests, and litter bags have increased our understanding of their ecological importance. Recent advances of molecular tools provide information on enzyme activities of microorganisms which interact with earthworms. Isotope labeling techniques have been used to quantify materials exchanged between earthworms and soils or atmosphere, and between earthworms and microorganisms. These new techniques are powerful but still limit our knowledge on the soil biogeochemical processes regulated by earthworms because they are indirect and static methods. In combination with existing methods, additional development of the tools to quantitatively and mechanistically evaluate their movements and exploring factors controlling their feeding behavior would be helpful to further understand their ecological roles. Such information will also enable us to establish more realistic models of soil ecosystems because many of the current models do not take the impact of soil animals into account [5]. Thus, observing earthworm feeding behavior, such as their response to some stimuli (such as light, temperature, vibration, and odor cues of food) in vitro without soil will be of importance albeit under very simplified artificial condition. The understanding of their physical activities resulting from their feeding behavior will advance quantification of the earthworm's contribution to whole below-ground biogeochemical processes. To determine earthworm activity, it is first necessary to quantify the extent to which they move and stretch their bodies and relate this to gas emissions from the soil. To confirm the activities, therefore, direct observation is a powerful tool and the combination of conventional methods with direct observation such as using a web camera or scanner will be of great merit in this field.

In addition to the importance of earthworms in ecosystem functions, they are also regarded as an important biological indicator of chemical toxicity in the soil. Ecotoxicology has traditionally used biological indicators to quantify or predict the changes in soil “health” or “quality” [5]. To date many studies have found serious effects of chemicals and heavy metals on the reproductivity, growth rate, and mortality of earthworms ([7] [8], [9]), and experimental protocol and earthworm culture techniques in vitro have been developed [10]. However, the observation of earthworms in response to such chemical pollution has been conducted over a relatively long time span with a lower resolution time (on the scale of minutes), and less attention has been paid to the signs in early stages, such as irregular behavior or morphological alterations induced by the uptake of pollutants. However, since the sudden changes in their activity and features might be also tightly connected to the damages and types of the pollutant, observations at high time resolutions (on the scale of seconds) should also be conducted.

To date, automated systems have been used to track movement with high time resolution continuously using a web camera for nematodes, ants, honeybees, and drosophila, and to subsequently analyze the continuous image data [11] [12] [13] [14] [15] [16] [17]. Image analysis is an efficient method for quantifying the tracking data. Previous studies have attempted to track earthworms directly in the soil by means of X-ray or minirhizotron [18] [19]; the time resolution, however, was low to track their dynamic changes over a long time period on a time scale of seconds. Revealing their habits without soil but with a high time resolution would be a first step in investigating their behavior. However, those tools cannot be applied directly to earthworms as some of their traits are different from other animals in terms of the extent to which they can stretch their bodies (their body stretch can reach 1.5 imes that of their default body length) [20].

A number of automated tracking systems for a model worm, *Caenorhabditis elegans*, have not considered changes in the extent of body stretch [20]. The WormScan is indeed a useful tool to observe population dynamics such as fecundity, reproductive activity, or mortality along with the growth rate that occur over a long time span. However, we still lack a program that can track dynamic change in their activities to observe a fast response to chemical pollutants, environmental changes or toxic substances. WormScan's default settings are for the nematode as a target animal. The features of nematodes, such as the movement and body size, vary widely from those of earthworms, therefore it requires intensive modification of the parameters of the program to apply it to earthworms. In addition, a standardized tracking system is required so that reliable comparisons can be made between species or phenotypes when tracking is done by different users. Standardized measurements will reduce misestimation and will output more reliable quantification.

Although existing methods such as WormScan [20] are candidates that may have been applicable in this study, the current WormScan program, which was originally developed for tracking nematodes, was not capable of following the changes in morphology of earthworms because earthworms move more quickly and over a wide area compared with nematodes. WormScan has been developed for observing population dynamics such as reproductive activity, fecundity or mortality which occur over a longer term span (several days to weeks), and the behavior is also relatively static. WormScan, therefore does not suit observations at a continuous and high time resolution, which captures dynamic and rapid movements. This is a remarkable property of earthworms and is an important factor in quantifying their physical activity. We believe that WormScan can track some objects of varying sizes, however we found that earthworms' movements are too fast and dynamic, and WormScan could not cope with this. The observation of dynamic changes would help to understand the ecological background of earthworm behaviors; chemical assays in vitro without soils could also be conducted. The existing application ImageJ (Fiji) (http://rsbweb.nih.gov/ij/version 1.46 for Windows) can provide a plug-in to manually track the two-dimensional positions and lengths of objects. Even when using this application, analyzing large data sets is labor intensive; therefore, a more reliable and automated quantification method is required for continuous image data.

In the present study, we developed a high-throughput program that tracks two-dimensional coordinates of multiple points and body lengths of objects in continuously acquired image data from earthworms; to the program was able to process approximately 1 frame per second. Following user initialization, the program tracks the head, central position, and tail of a single earthworm. This program provides a means to track animals that change their body lengths over time both accurately and efficiently, though still with limited performance. The output was subsequently used to calculate the velocity of those points. Finally, in the future, the quantified behavior could be incorporated into soil material flow dynamic models or applied to measure their reactions to chemical inputs in the soil.

## Materials and Methods

### Study animals

Two different species of earthworm were used in this study; *Eisenia japonica* [21] with a body size ranging from 4–17 cm [22], and *Metaphire hilgendorfi* [21] with a body size ranging from 9–30 cm [23]. The lifespan of both is usually 6 months from spring to fall. Both species are common in Japanese mountains and fields, and both genera have a wide global distribution [24]. *M*. *hilgendorfi* is epigeic because it primarily inhabits the litter layer and *E*. *japonica* is polyhumic-endogeic because it mainly lives in the topsoil layer and has more inorganic content in the gut [25].

### Continuous recording of earthworm movement in glass containers (image capture)

**Figure 1:**
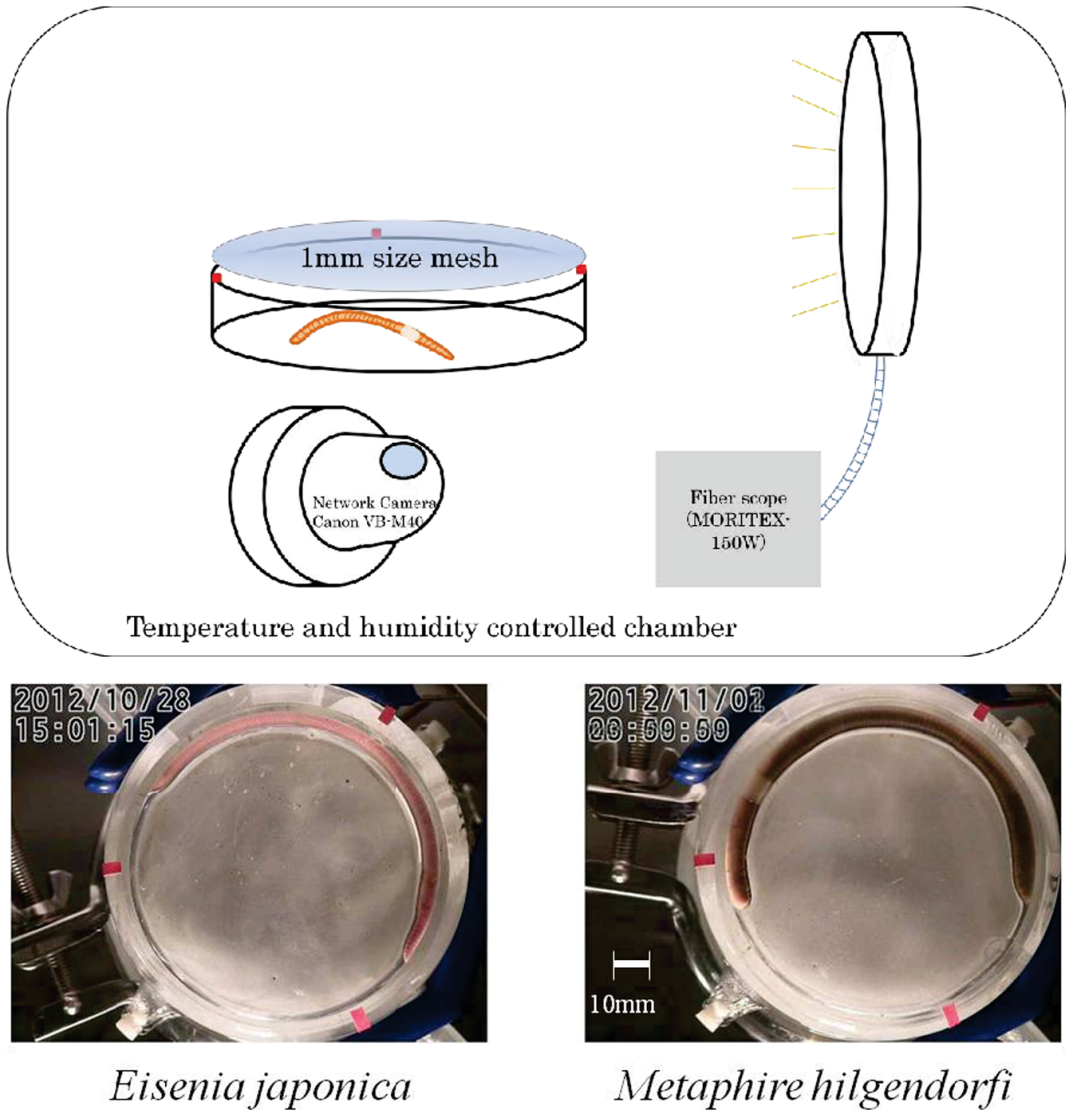
Webcam setup and study animals. The upper diagram shows the system for webcam recording in a controlled chamber. The earthworms used in this study are shown in the bottom panel. The diameter of the glass Petri dish was 85(inside diameter) and the height of the Petri dish was 20 mm.

Adult earthworms were captured from a field at the National Institute for Agro-Environmental Sciences, Tsukuba, Japan on 28 October, 2012. Earthworms were kept in a plastic box (inner diameter 15 cm, height 6 cm) with approximately 100 g of alluvial soil at 60% of its water holding capacity until the experiment. The weights of *E*. *japonica* and *M*. *hilgendorfi* used in the experiment were 1.45 and 4.75 g, respectively. *E*. *japonica* and *M*. *hilgendorfi* were transferred from the plastic box to glass containers for network-camera recording inside an incubator, where the mean ambient temperature was set at 15°C and the ambient air humidity was saturated at the given temperature (Fig. 1). A fiber scope light was used to maintain constant light conditions (MHAB-150W, Moritex, Tokyo) at approximately 500 lux outside of the container and app. 250 lux inside the container; this was measured with a radiometer (T-10A, Konica Minolta Optics, Inc., Tokyo, Japan). The glass containers were covered with 1-mm mesh plastic cloths and fixed with double-sided cellophane tape to prevent the earthworms from escaping during the experimental period. A small fan (OD2510, Orion Fans, Dallas, USA) was placed on the top of the glass container to ventilate the air inside and wet tissue was put in to keep the humidity constant during the measurement period. A network camera (VB-M40, Canon Inc., Tokyo, Japan) was fixed to the bottom of the glass container with approximately 60 cm of distance between the camera and the glass container (Fig. 1). The network camera was fixed in parallel to the glass container firmly while capturing the image. The position of the camera was calibrated using the ruler to ensure the accurate pixilation of the actual length. The network camera uses a lens that is 40 mm in diameter and can cover 60.4° from the horizontal angle. The captured image data was 640×480 pixels in size. To obtain the coordinates of the glass container, marks were fixed at three positions on the edge of the containers with red tape. The round glass containers were 85 mm in inside diameter and 2.5 mm in thickness. The image data were recorded as a movie using a network camera continuously recording every second and saved as JPEG files. One pixel was equivalent to 0.2 mm in the captured image data.

To estimate local image distortions, checkerboard (8 rows and columns) was located for image capturing system on the same position as the glass container. The length of the diagonal lines were measured using a vernier caliper with a 0.1 mm precision and calculated the errors. The maximum error in mm was 0.2 mm that resulted the precision less than 0.1%.

### Image analysis

Two methods were implemented: a manual tracking method and a new automated tracker. First we used a manual tracking method relies on the Fiji implementation of ImageJ (http://rsbweb.nih.gov/ij/version 1.46 for Windows) with an additional plugin, Manual Tracking (http://fiji.sc/wiki/index.php/Manual_Tracking). Currently manual tracking is a standard method to track animal behavior using image data. Second, we then used a newly developed program (MimizuTrack) that was compared with Manual Tracking in Fiji.

### Manual tracking

Using the Manual Tracking plug-in on the Fiji implementation of ImageJ, head and tail of the body were tracked separately. The static image data (JPEG) captured at 1 second intervals were converted to movie data, using a function in Fiji to convert from JPEG into.avi formatted data. Each coordinate per one second was tracked to obtain the velocity of the movement. We then handled the output data as ground truth for further evaluation since the data were input by expert users. The output data were used for evaluation of the program described in the next section. Our program was benchmarked against user input only. This was because no automated tracking system currently exists that is directly comparable to our tracker in this specific setting.

### Automatic tracking - Initialization Process

**Figure 2:**
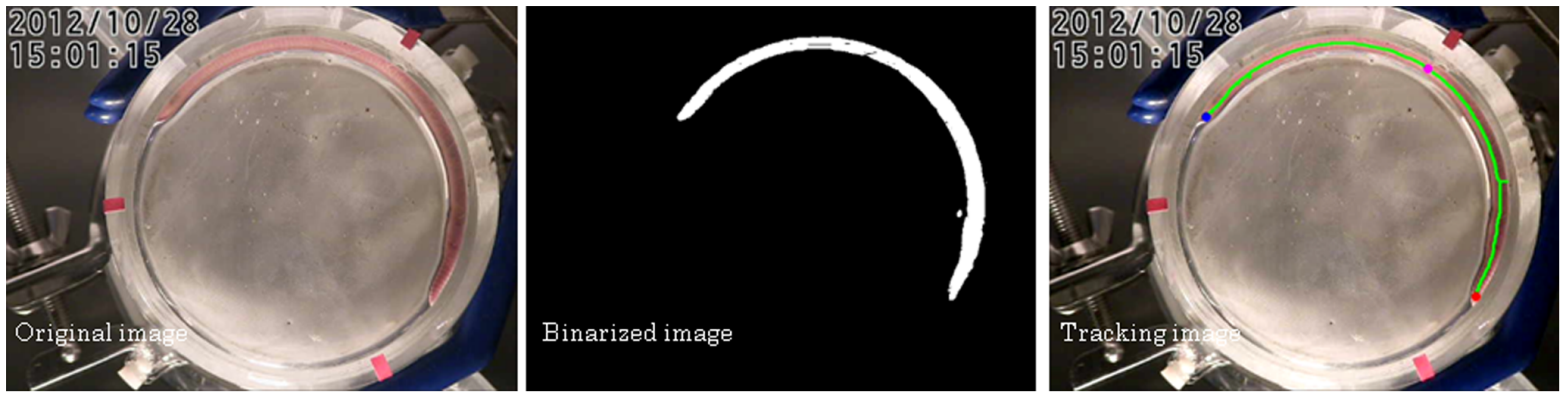
Earthworm body thinning procedure and determination of the head, tail, and central points. The captured image data were used for the latter.

**Figure 3:**
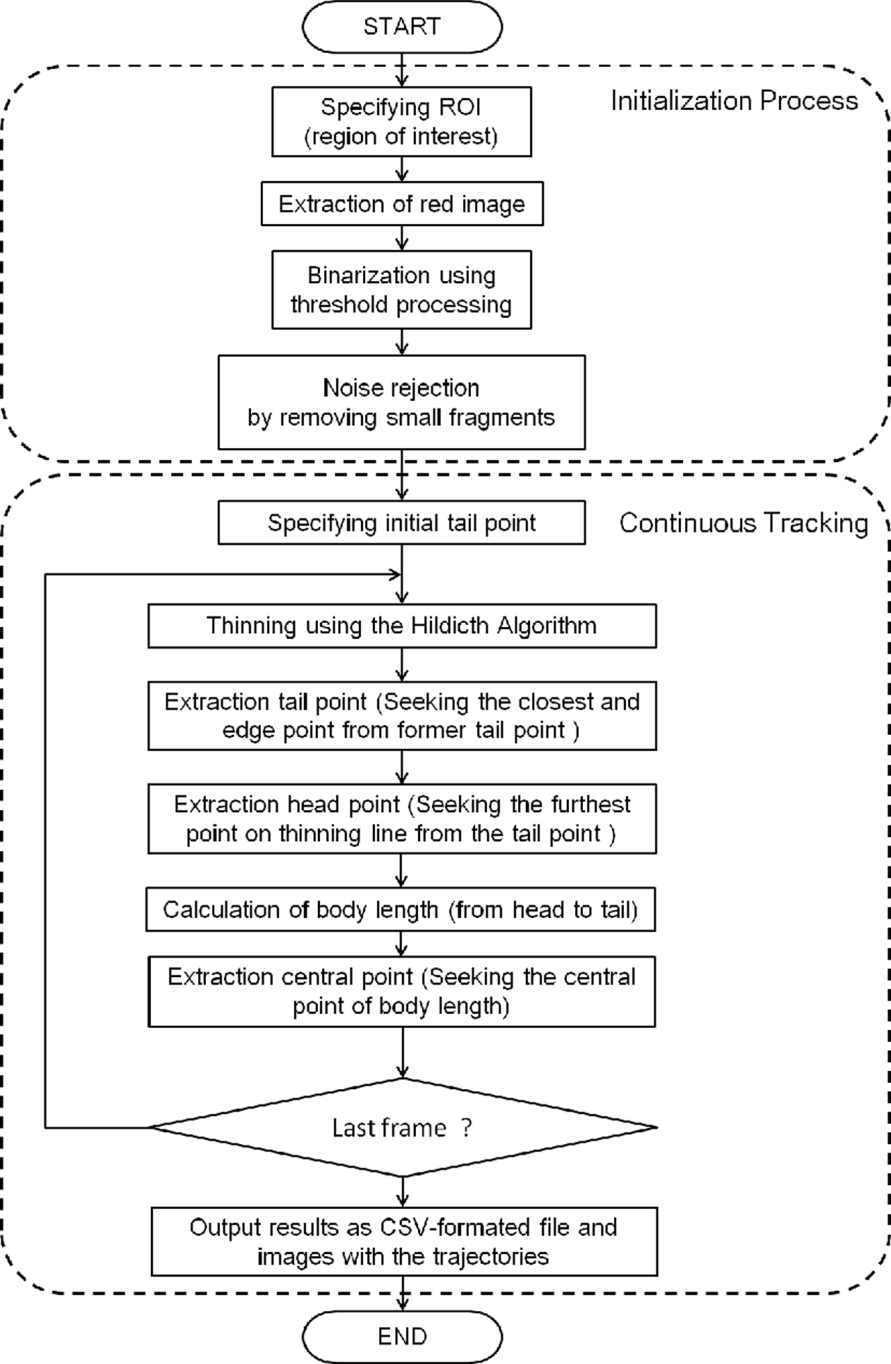
Workflow of the proposed method. Our method consists of two processes; ‘Initialization Process’ and ‘Continuous Tracking’.

The image data obtained were first processed to separate the objects (earthworms from the background). In the original image, the contrast between the earthworms and the background was highest in the red channel so only this component was used for the process. The procedures are done manually as an initial step, then the image data are processed using a program (image shown in Fig. 2); We developed several programs to track moving objects over time, such as honeybees or plant roots in previous studies [12] [26] [27] the source code for which is original, and we have developed a new program (MimizuTrack) for earthworms based on the previous programs. The steps are shown in Fig. 3 and detailed below. The detailed initialization process are described as follows:

- The outline of the inside of the glass container was determined using a tool for drawing around the shape and cutting the outside of the image.
- A color threshold was determined to highlight the color of the earthworm body and split the color channels into three colors (green, blue, and red). The red image was then used for further analysis. Earthworms were assigned a value of 255 and the non-earthworm areas were eliminated by applying a value of 0.
- A threshold to extract the earthworm body was determined and a binary image with white for the object and black for the background was generated.
- The noise was eliminated by removing small fragments from the background.

### Automatic tracking - Continuous Tracking

The rest of the image processing was carried out by our newly developed program. The procedures are shown in Fig. 2. We developed a prototype program using Microsoft Visual Studio 2010 (Visual C++2010) and OpenCV 2.31 on a computer with an Intel Core i5 2.50 GHz (CPU), 16 GB (Memory), 640 GB (HDD), and running Microsoft Windows 7 Enterprise 64-bit (OS). Our developed software is executed as a Win64 console application on Windows.

The tracking method is as follows;

- Thinning the earthworm body areas

To extract feature points, such as the head, tail, and center points, the program was tasked with identifying the end-points (tails) of the line segment corresponding to the worm body. That is as thin as possible while remaining fully connected and centered. Using binary images of the earthworm body, the program was able to obtain three feature points on each frame. First, the Hilditch algorithm is used to reduce the pixel region representing the earthworm to its skeletal form [28]. The end-points of the center line are recognized as candidates of earthworm feature points.

- Extracting the head, tail, and center point of individuals (feature extraction)

The next step was to determine the head, tail, and center point. The program extracted the feature points of the earthworm body by first identifying the head and tail using the different features between the two points (tail points always move slower than the other points), then the center point was determined. To initialize the program, the tail point was determined manually. By doing this, the program automatically calculated four-neighbor distances of the center line from the tail point and extracted the point at the furthest distance. The program defined this as the head point of the earthworm. Then, the program recognized the halfway point between the head and tail as the central point of the body. Using this process, the program could obtain the initial feature points of the earthworm (i.e. head, tail, and center points). From the second frame, the program calculated new feature points using the previous ones. First, the program extracted new tail points based on the fact that the tail points of the earthworm move slower than the other points. Using this feature, the program recognized the edge points as new tail points with the shortest distance from the previous points. Both head and center points were obtained by the same procedure, which were extracted from the initial head and center points. These processes were carried out on all of the images. The program outputs positional data in each frame to a CSV file, allowing trajectories over time to be visualized.

### Statistical Analysis

All statistical analyses were performed using SPSS 10.05 (SPSS Inc., Chicago, IL, USA). Relationships between two variables (program output and manual output) were assessed using standard bivariate correlation procedures. The correlation coefficient is shown in the text below and the figures.

## Results

### Evaluations of automatic output using the manually tracked values

#### Tracking and extraction of each point

**Figure 4:**
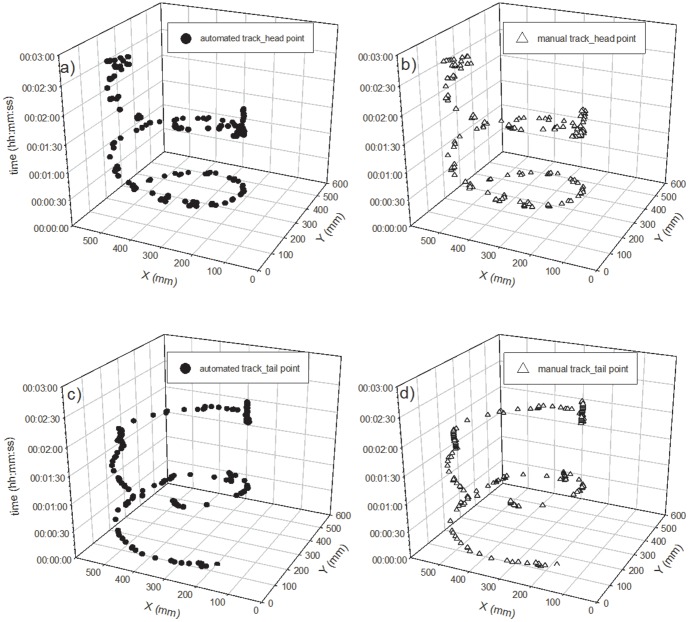
The automatically and manually measured two-dimensional coordinates of *Eisenia japonica* over time. Each panel shows the head point in the upper panel and the tail point in the bottom panel.

**Figure 5:**
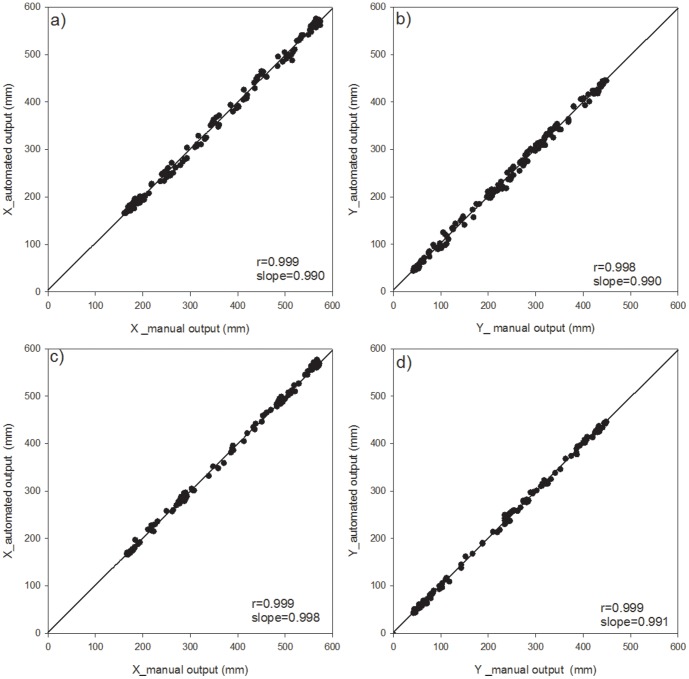
Correlations between the automatically and manually measured two-dimensional coordinates of *Eisenia japonica*. Each panel shows the head point in the upper panel and the tail point in the bottom panel.

**Figure 6:**
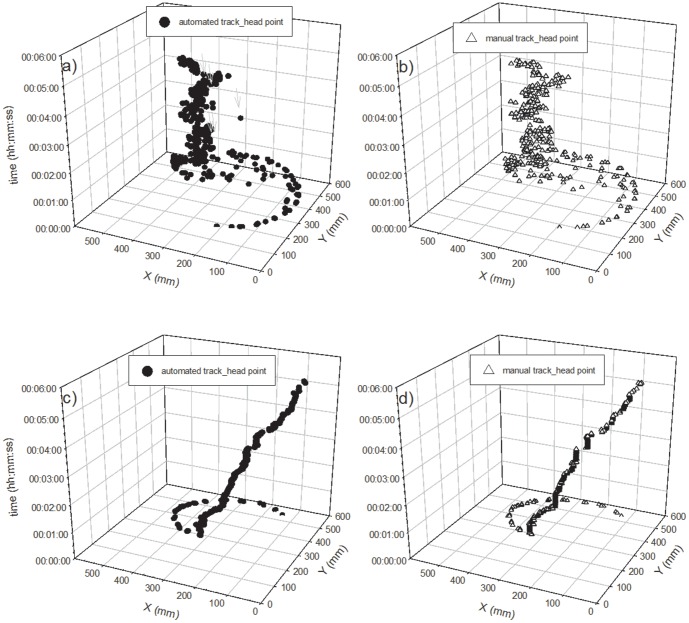
The automatically and manually measured two-dimensional coordinates of *Metaphire hilgendorfi* over time. Each panel shows the head point in the upper panel and the tail point in the bottom panel.

**Figure 7:**
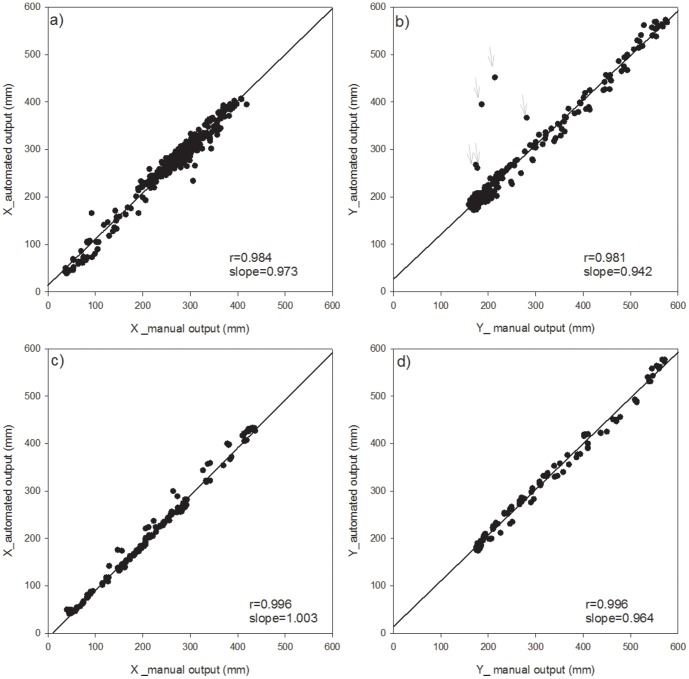
Correlations between the automatically and manually measured two-dimensional coordinates of *Metaphire hilgendorfi*. Each panel shows the head point the in upper panel and the tail point the in bottom panel.

**Figure 8:**
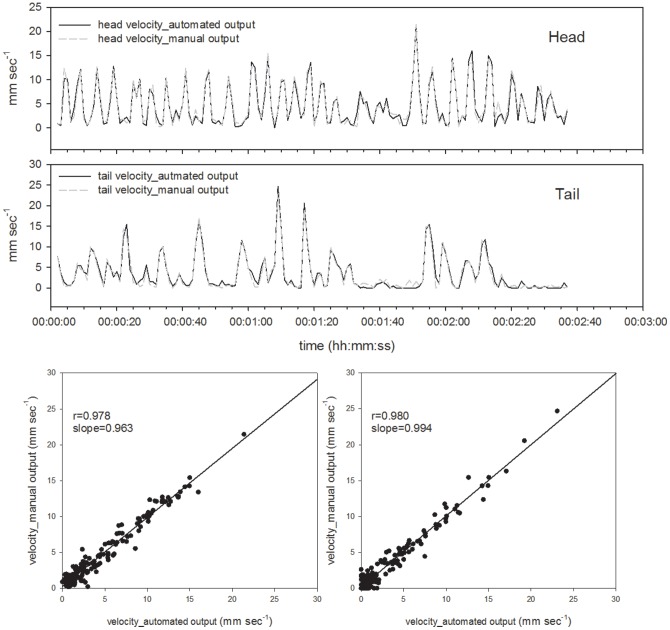
Comparison of the automatically and manually calculated velocity of each point of *Eisenia japonica*. The upper panel shows the calculated velocity of the head point and the central panel shows that of the tail point. Correlations between the automatically and manually calculated velocities of *Eisenia japonica*.

**Figure 9:**
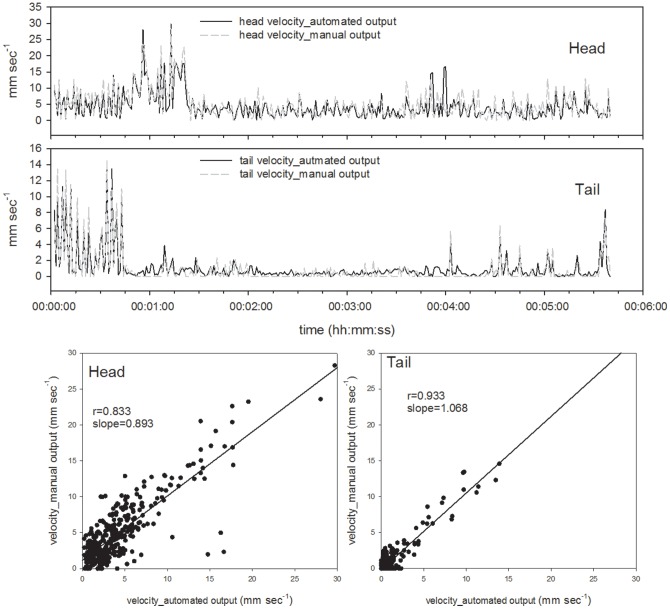
Comparison of the automatically and manually calculated velocity of each point of *Metaphire hilgendorfi*. The upper panel shows the calculated velocity of the head point and the central panel shows that of the tail point. Correlations between the automatically and manually calculated velocities of *Metaphire hilgendorfi*.

**Figure 10:**
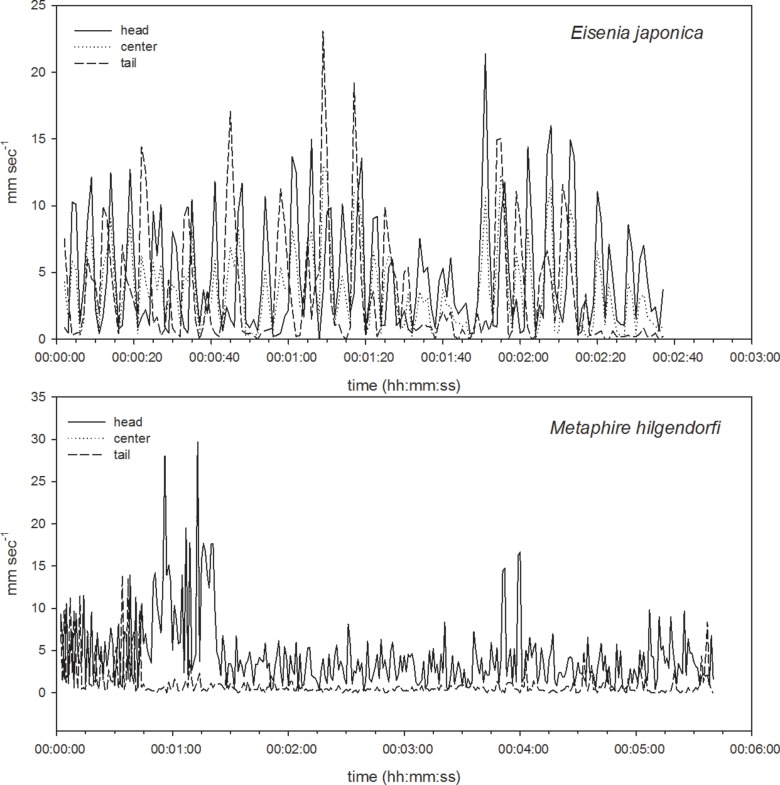
Comparison of velocities among different points (head, central, and tail points). The upper panel shows the velocities of each point of *Eisenia japonica* and the bottom panel shows those of *Metaphire hilgendorfi*.

**Figure 11:**
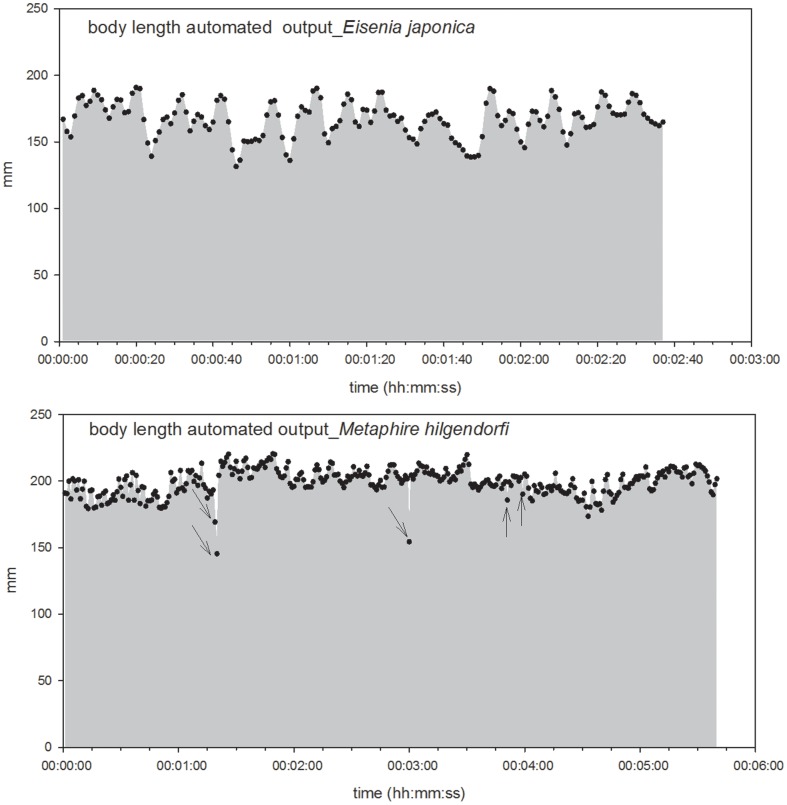
Change in body length over time. The upper panel shows the length of *Eisenia japonica* and the bottom panel shows that of *Metaphire hilgendorfi*.

First, we were successful in tracking the three target points, (the head, center, and tail of the body) on a time scale of seconds, continuously followed by body length calculations using the newly developed program. We used a part of the obtained data for the evaluation of the program which shows typical movement of two species of earthworms every 150 seconds and 300 seconds for *E. japonica* and *M. hilgendorfi* respectively (Figs. 4–11). The tracking of the head and tail with the automatic and manual programs over time are shown in Figs. 4 and 6 respectively. All of the regression lines between the automatically and manually tracked coordinates were close to 1 (>0.98), especially in the case of *E. japonica* (Fig. 5). In contrast, some outliers were observed in *M. hilgendorfi* as indicated by the arrows in Fig. 7. There were no significantly different measurements observed in *E. japonica* between different body parts; however, significant differences were observed between the different body parts of *M. hilgendorfi*. In general, the errors in measurement of the head point (r = 0.981, r = 0.984, X and Y coordinates, respectively) were larger than those for the tail point (r = 0.996, r = 0.996, X and Y coordinates, respectively). One reason for this was that the head moved more frequently while the tail stayed in the same position. Since the program tracks and determines the feature points by predicting the next steps, the program may fail to track the objects if the objects (in this case, the heads) move more broadly in the image-capturing interval (1 second). In the case of *M. hilgendorfi*, when they looped, coiled, or crossed their bodies, the program faced difficulties in recognizing the edges, and failed to detect the head positions.

#### Calculations of velocity of each point and body length

Using their coordinates, the velocity of each point was calculated. The output data obtained with the automated program were further confirmed by comparing them with the manually acquired data (Figs. 9 and 10). The accuracy of each output was satisfactory for the application to quantify earthworm activity over time. Some outliers in head velocity in *M. hilgendorfi* were due to the misestimation of head points by the automated program, in which case the head was attached to a part of the body. Otherwise, the automated program satisfactorily detected the peaks in terms of timing and absolute values. As shown in Table1, the mean velocities of the head and tail of *E. japonica* calculated by the automated program were 4.75±4.36 and 3.44±4.38 mm sec^−1^, respectively, while those from the manually calculated values were 4.81±4.29 and 3.56±4.44 mm sec^−1^, respectively. The mean velocities of the head and tail of *M. hilgendorfi* calculated by the automated program were 4.38±4.13 and 1.03±1.92 mm sec^−1^, respectively, while those from the manually calculated values were 5.09±4.43 and 0.92±2.21 mm sec^−1^, respectively. The differences between the automated and manually calculated values were 0.07±0.92 and 0.11±0.88 mm sec^−1^ for the head and tail of *E. japonica*, respectively and 0.71±2.49 and 0.11±0.80 mm sec^−1^ for those of *M. hilgendorfi*, respectively (Table2). The difference was largest in the head of *M. hilgendorfi* but the others matched quite well, with the differences being less than 0.2 mm sec^−1^. The tail points tended to move slower than the head points according to the calculated velocities, and the head point of *M. hilgendorfi* moved faster than that of *E. japonica* by 1.65±7.07 mm sec^−1^. The body lengths were calculated from the binary data, which were converted into a line (shown in Table3).

**Table 1:** Mean, maximum, and minimum values of automatically and manually calculated velocities of each point (head and tail) of two earthworm species (*E. japonica* and *M. hilgendorfi*).

Species	*velocity*	mean (mm sec^−1^)	max. (mm sec^−1^)	mini. (mm sec^−1^)
***E. japonica***				
Program	head	4.75±4.36	21.37	0
	tail	3.44±4.38	23.09	0
manual	head	4.81±4.29	21.45	0.21
	tail	3.55±4.44	24.69	0
				
***M. hilgendorfi***				
program	head	4.38±4.13	29.71	0.21
	tail	1.03±1.92	13.9	0
manual	head	5.09±4.43	28.29	0
	tail	0.92±2.21	14.58	0

The errors indicate standard deviations over time.

**Table 2:** Mean maximum values of differences between automatically and manually calculated velocities of each point (head and tail) of two earthworm species (*E. japonica* and *M. hilgendorfi*).

Species	mean (mm sec^−1^)
***E. japonica***	
head	0.07±0.92
tail	0.11±0.88
***M. hilgendorfi***	
head	0.71±2.49
tail	0.11±0.80

The errors indicate standard deviations over time.

**Table 3:** Mean, maximum, and minimum values of body length of two earthworm species (*E. japonica* and *M. hilgendorfi*).

*body length*	mean (mm)	max. (mm)	mini.(mm)
***E. japonica***	167.02±13.62	190.73	131.44
***M. hilgendorfi***	199.28±10.05	220.38	145.33

The errors indicate standard deviations over time.

The mean lengths of *E. japonica* and *M. hilgendorfi* were 167.02±13.62 and 199.28±10.05 mm, respectively, while the maximum and minimum lengths of *E. japonica* were 190.73 and 131.44 mm, respectively, and those of *M. hilgendorfi* were 220.38 and 145.33 mm, respectively. Therefore, the stretch from minimum to maximum was 59.19 and 75.05 mm for *E. japonica* and *M. hilgendorfi*, respectively. The extent of the stretchiness was in agreement with previously reported values [1], which indicated that the body could reach 1.5 times longer than its default length. Moreover, our program also generated the change in length over time per second, so that we could observe the frequency of the stretchiness for further applications (Fig. 11).

## Discussion

To date, there exists no method to process and extract such high time resolution image data (on a time scale of seconds) capturing three feature points along with tracking elastic objects such as earthworms, which move quickly and widely, although the program is still a prototype at this stage. A number of automated systems have been developed to track insects' movements, and our earthworm tracking system might be similar to that of *Caenorhabditis elegans* (one of the model nematodes) [20], [29]. Our program can deal with a time scale of seconds, though there remain some problems with the program. The program could not cope with irregular shapes of earthworms, such as coiling or overlapping. This could be achieved by modifying the program in the future using the Knot Theory, or topological concept. The program also failed to follow the feature points when the head moved more frequently than the general movement. Furthermore, in the case of *M. hilgendorfi*, when they coiled or crossed their bodies, the program faced difficulties in recognizing the edges, and failed to detect the head positions. To overcome such problems, one solution would be to obtain images at higher time resolutions so that the program could keep tracking more frequently moving objects without losing the target points because the higher time resolution is closer to continuous data. Increasing the contrast between the background and the objects for image analysis would also achieve more precise results. Furthermore, there would be alternative methods to detect feature points such as differentiating the shapes or brightness of head or tail, which will further improve the program.

The velocity of each point of the different body parts of *E. japonica* was synchronized; in contrast, those of *M. hilgendorfi* were not. In the case of *M. hilgendorfi*, the head and center points were synchronized but the tail point was not; it tended to stay still on the same coordinate. We recognize that the velocities were under non-soil conditions; therefore, earthworms in the soil will be much slower than our calculations indicate. However, the program picked up the features of each species when observed under the same conditions. We consider our newly developed program to be sufficient to measure earthworm activity; this method can be used to compare dynamic changes in these parameters between species or in response to environmental variables such as temperature, light, and moisture, as well as chemical contaminants.

Image data is powerful, direct information that can be used to quantify physical information of changes in earthworm's activities. The intention of this study is to apply the program to quantify the physical parameters to indicate earthworm's activity from their movement speed or body stretchiness. It will be more beneficial if these data are combined with other data, such as data on gas emissions from the body, enzyme activities using molecular tools, or pathway of elements using isotopic data for mechanistic understanding. The method used in this study is artificial; a small Petri dish without soil as used. However, this study will advance our understanding of the basic behavior of earthworms depending on the alteration of their living environment. This basic behavior can be incorporated in models in terms of material flow belowground or reactions in response to chemical contaminant in soil [5].

Combining different kinds of studies will help to unravel or predict the complex processes in belowground systems [6]. Furthermore, although this system could not be applied to observe their behavior directly in the soil, culturing earthworms in transparent gels or agar medium would be a useful method to observe them three-dimensionally. Furthermore, applying other rapidly developing state of the art technologies, such as MRI or radar, to observe earthworms under soil conditions, it will be theoretically possible to directly observe their behavior in soil, though the time resolution will not be high enough. In fact, radar is already being used for root growth observations in soil.

## Conclusions

We demonstrated that our newly developed program successfully tracks the movement of two species of earthworms that have different features in terms of body size, mass, and morphology as well as movement. Based on our data, the program reduced the time required for manual tracking 3-fold and the program could track the movement which is inherent in earthworm behavioral ecology studies. Large continuous image data sets can be immediately processed and the program can determine earthworm body parts along with their changes in elastic body lengths over time. In the future, quantified earthworm behaviors could be incorporated in material flow dynamic models in soil or in models of the response of earthworms to chemical inputs to soil.

The software “MimizuTrack” will be released on INCF Software Center (http://software.incf.org/) under the BSD license. Otherwise the program is open upon request to the following contact person; Assis. Prof. Toshifumi Kimura (email: kimura@shse.u-hyogo.ac.jp).
